# Whole Exome Sequencing Identified *MCM2* as a Novel Causative Gene for Autosomal Dominant Nonsyndromic Deafness in a Chinese Family

**DOI:** 10.1371/journal.pone.0133522

**Published:** 2015-07-21

**Authors:** Juanjuan Gao, Qi Wang, Cheng Dong, Siqi Chen, Yu Qi, Yuhe Liu

**Affiliations:** 1 Department of Otolaryngology, Head and Neck Surgery, Peking University First Hospital, Beijing, China; 2 Department of central laboratory, Peking University First Hospital, Beijing, China; University of Heidelberg, GERMANY

## Abstract

We report the genetic analysis of autosomal dominant, nonsyndromic, progressive sensorineural hearing loss in a Chinese family. Using whole exome sequencing, we identified a missense variant (c.130C>T, p.R44C) in the *MCM2* gene, which has a pro-apoptosis effect and is involved in the initiation of eukaryotic genome replication. This missense variant is very likely to be the disease causing variant. It segregated with hearing loss in this pedigree, and was not found in the dbSNP database or databases of genomes and SNP in the Chinese population, in 76 patients with sporadic hearing loss, or in 145 normal individuals. We performed western blot and immunofluorescence to test the MCM2 protein expression in the cochlea of rats and guinea pigs, demonstrating that MCM2 was widely expressed in the cochlea and was also surprisingly expressed in the cytoplasm of terminally differentiated hair cells. We then transiently expressed the variant MCM2 cDNA in HEK293 cells, and found that these cells displayed a slight increase in apoptosis without any changes in proliferation or cell cycle, supporting the view that this variant is pathogenic. In summary, we have identified *MCM2* as a novel gene responsible for nonsyndromic hearing loss of autosomal dominant inheritance in a Chinese family.

## Introduction

Hearing loss is the most common form of sensory impairment in humans. About half of the occurrences of hearing loss are caused by genetic factors. Hereditary hearing loss can be subdivided into nonsyndromic hearing loss and syndromic hearing loss according to the clinical manifestations [1–3]. Syndromic deafness has abnormalities in other parts of the body, and accounts for about 30% of hereditary hearing loss. In contrast, no symptoms or signs are found in other parts of the body in nonsyndromic hearing loss, which accounts for about 70% of hereditary hearing loss [2]. In nonsyndromic hearing loss, 75–80% of patients show an autosomal recessive inheritance and 18% of patients are of autosomal dominant inheritance [2, 4]. To date, more than 70 loci have been identified in about 30 genes encoding various proteins with various functions in the auditory system and responsible for autosomal dominant deafness (*http*:*//hereditaryhearingloss*.*org*). In general, these genes are involved in hair bundle morphogenesis [5], form constituents of the extracellular matrix [6], play a role in cochlear ion homeostasis or serve as transcription factors [7]. With the rapid development of second generation sequencing technology, new genes accounting for hereditary hearing loss have been identified in recent years, such as *TBC1D24*, *P2RX2* [8] and *TNC* [9].

In this study, using whole exome sequencing, we have identified the minichromosome maintenance complex component 2 gene (*MCM2*) as a novel gene causing autosomal dominant progressive sensorineural hearing loss in a large Chinese pedigree [10–13]. The protein MCM2 encoded by *MCM2* is a member of eukaryal MCM2–7 subunits, which is well known to be essential for regulating DNA replication and is involved in a variety of chromosome transactions [14]. The MCM2–7 proteins can be arranged in a hexaheteromeric complex working as a eukaryotic DNA helicase. They play essential roles in the initiation and elongation of DNA synthesis and the assembly and activation of the pre-replicative complex [15–17]. MCM2, as an independent protein, seems to have a more general function. The N terminal of MCM2 interacts with RNA polymerase II, MYST family members and others [14, 18–20]. Moreover, MCM2 has also been proven to have a pro-apoptotic effect lately. An elevated level of MCM2 in cytoplasm is involved in the P53-dependent apoptosis signaling pathways through its interaction with gp70 [21, 22]. In vitro analysis revealed that MCM2 over-expression induced apoptosis in HL60 cells [23].

In this paper, we explored the expression pattern of MCM2 in the terminally differentiated hair cells of cochlea and the results consisted with the previous conclusion that a minor level of MCM2 was expressed in hair cells by DNA microarray [24]. We also tentatively detected effects of variant MCM2 in cell apoptosis, cell proliferation and cell cycle phases in HEK293 to pursue mechanism of this variant gene leading to hearing loss.

## Materials and Methods

### Genomic DNA sample collection

The study on this Chinese family with hearing loss (Family #51) was approved by the Medical Ethics Committee of Peking University First Hospital. All participants signed the informed consents. The 13-year-old child’s written consent was obtained from his parents. Sensorineural hearing loss diagnosis was performed via the standard audiometry in a sound-proofed room according to the clinical standards. Blood samples were collected from all available family members in this pedigree, as well as 76 patients with sporadic hearing loss and 145 normal individuals as controls at the Department of Otolaryngology and Head-neck Surgery, Peking University First Hospital. Genomic DNA was extracted using the Qiagen blood DNA extraction kit (Qiagen, Hilden, Germany).

### Whole exome capture and library construction

Human exome capture was performed following the protocol from Illumina’s TruSeq Exome Enrichment Guide (Illumina, San Diego, CA, USA). Illumina’s TruSeq 62 Mb Exome Enrichment kit was used as exome enrichment probe sets, and 5 μg of genomic DNA in 80 μL of Buffer EB (Qiagen) was fragmented in a Biorupter UCD-200(Diagenode, Belgium) to sizes of 100–500 bp. DNA concentration was estimated by OD_260_ measurement and quantitative real-time polymerase chain reaction (PCR) analysis. Captured DNA libraries were sequenced with the Illumina HiSeq 2000, yielding 200 (2 × 100) base pairs from the final library fragments using V2 reagent. Base calling was performed with CASAVA 1.8 software (Illumina). The reads were aligned with the human genome reference sequence (UCSC hg19) using the Burrows-Wheeler Alignment (BWA) tool, version 0.5.9rc1. Variants (SNPs and indels) were called with vcftools of SAMTools software version 0.1.16 [22]. High VarQuality SNPs were annotated with Perlscript into functional categories such as missense, nonsense, splice sites, coding, non-coding and untranslated regions (UTRs). Amino acid substitution where an amino acid substitution affects protein function was annotated with SIFT (Sorting Intolerant From Tolerant) and PolyPhen-2 (Polymorphism Phenotyping v2).

### Identification of the candidate variants

After analyses of the results from whole exome sequencing, we considered first the 10 candidate variants which were probably significant in autosomal dominant deafness in this pedigree (see Results section). PCR sequencing was performed using 10 pairs of primers aiming to confirm these variants in the family members of this pedigree. The forward primer 5′-AATAGGCAAATGACATAACC and reverse primer 5′-GTGCTCAAAGGCAAGAAT were used for the amplification of exon 2 of the *MCM2* gene in the 13 family members of this pedigree, 76 patients with sporadic hearing loss, and 145 normal controls. PCR products were directly sequenced using an ABI 3730 sequencer.

### MCM2 mRNA identified by RT–PCR in the inner and outer hair cells of guinea pigs

The animal experiments were performed in accordance with the recommendations in the Guide for the Care and Use of Laboratory Animals of Peking University. The protocols were approved by the Committee on the Ethics of Animal Experiments of the Peking University First Hospital (Permit Number: J201430). All efforts were made to minimize suffering. After anesthesia with pentobarbital, two Albino guinea pigs (250 g) were decapitated and the temporal bones and otic capsules were removed. The remaining parts were soaked in a RNA stabilization solution (Ambion, Life Technologies) to remove the volute. Total RNA was extracted from the cochleae with Trizol (Life Technologies) and reverse-transcribed using the Reverse Transcript system (A3500, Promega); PCR analysis was carried out using a GoTaq Hot Start Green Master Mix (M5122, Promega). Specific primers were designed using Primer3 Plus software based on the MCM2 mRNA sequence of guinea pig in GenBank (XM_003461494.2). The forward primer was 5′-TGGGACTCACGGCCTATGTA and the reverse primer was 5′-CTCCTTTCGCAGGTCACTGT with the expected PCR product of 596 bp. PCR without the cDNA was always used as the negative control. PCR products were directly sequenced to confirm the accuracy.

### MCM2 expression in inner ears identified by western blot

Cochleae from two Albino guinea pigs were ground in lysis buffer (50mM Tris HCL, pH 7.4, with 150mM NaCl,1mM EDTA, and 1% TRITON X-100) supplemented with protease inhibitor cocktail (M221, AMRESCO) to produce cochlear total protein. Cochleae from an additional four Albino guinea pigs were dissected into one part containing the spiral ligament and stria vascularis and another part containing the modiolus and basilar membrane, and tissue lysates were extracted from both parts. After separation of these protein lysates in SDS–PAGE and transfer of the proteins onto nitrocellulose membrane, the membrane was blotted with 1:1000 rabbit anti-MCM2 antibody (#3619, Cell Signaling) diluted in 5% non-fat milk/Tris-based saline/0.05% Tween 20 solution. The blotted antibody was recognized by 1:10,000 peroxidase-conjugated mouse anti-rabbit IgG antibody, and developed with the enhanced chemiluminescence method (ECL; Immobilion Western Chemiluminescent HRP Substrate, Millipore).

### MCM2 expression in rat inner ears identified by immunofluorescence

For the dissection, the otic capsules were dissected from three Sprague–Dawley rats (6–8 weeks) and soaked in phosphate-buffered saline (PBS) to obtain the organ of Corti. After removal of the tectorial membrane and stria vascularis, the sensory epithelium (organ of Corti) was separated from the modiolus with a pair of tweezers. The dissected cochlear sensory epithelial layer was fixed in 4% paraformaldehyde, washed several times, and incubated in a blocking solution (10% goat serum/PBS/0.25% Triton X-100) for 1 h. The sensory epithelial layer was then incubated with 1:500 rabbit anti-MCM2 antibody (#3619, Cell Signaling) or goat anti- MCM2 antibody (sc-9839, Santa Cruz Biotechnology) in the blocking solution at 4°C overnight. In the control experiment, the anti-MCM2 antibody was omitted. After washing, the sensory epithelial layer was incubated with 1:200 Alexa Fluor 488-conjugated goat anti-rabbit IgG (A11034, Molecular Probes) or donkey anti-goat IgG (A11055, Molecular Probes) in PBS at room temperature for 1 h. After washing for 1 h, the sensory epithelial layer was carefully spread on a slide and mounted with fluorescent mounting medium containing 4',6-diamidino-2-phenylindole (DAPI). The slides were then observed with a confocal microscope. Cochlea frozen sections from Sprague–Dawley rat (6–8 weeks) were performed as the following: after removal of the stapes from the oval window and piercing of the round window, 4% paraformaldehyde fixative was perfused gently through the cochlea. Inner ears were immersed in fixative for 24h in 4°C, followed by decalcification in 120mM Ethylene Diamine Tetraacetic Acid (EDTA) for 1 week at room temperature, dehydration in 20% sucrose solution for 24h, and embedded in opti-mum cutting temperature compound (OCT). Serial sections were obtained at 8μm thickness and used for immunofluorescence. The immunofluorescence procedure was the same as dissection.

### Transient expression of wild-type or c.130C>T variant MCM2 in HEK293 cells

A mammalian expression plasmid containing an open reading frame of human MCM2 cDNA with a green fluorescent protein (GFP) tag at the 3′ end was obtained from Origene (Rockville, MD, USA). We also used a site-directed mutagenesis kit (Transgen, Beijing, China) to produce the c.130C>T variant in MCM2 cDNA in the plasmid. The sequences in the wild-type and variant plasmids were confirmed by sequencing.

We then used human kidney cell line HEK293 cells to examine the role of variant MCM2 in these cells. HEK293 cells were cultured in Dulbecco’s modified Eagle’s medium (DMEM) supplemented with 10% fetal bovine serum (FBS). The cultured cells were divided into four groups, treated with liposome transfection reagent only (lipo group), empty vector transfection (empty vector group), wild type MCM2 plamid transfection (WT group), and variant type MCM2 plasmid transfection (M group) respectively. The first two groups were used as controls and we mainly focused on the differences between wild type and variant type MCM2 expressed cells. For transfection, 2 μg of plasmid DNA in 200 μL DMEM was mixed with 6 μL liposome transfection reagent (MegaTran 1.0, Transgen, Beijing, China), incubated at room temperature for 10 min, and added to 5×10^5^ HEK293 cells in 1.5 mL of culture medium. After 48 h, about 80% of the cells expressed green fluorescence, indicating that the transfection efficiency was about 80%. The cells were then processed for flow cytometry, western blot and proliferation test.

### Apoptosis assay

After being treated for 48 hours, both adherent and non-adherent cells were harvested, stained with annexin-V-PE and 7-AAD using an apoptosis assay kit (keyGEN, Nanjing, China), and assayed in a Beckman Coulter flow cytometer. Cells were gated for apoptosis assay. The experiment was repeated three times. The cell lysates were also prepared for western blot. Caspase-3 and cleaved caspase-3 antibodies (ab32351,abcam; #9661, Cell Signaling) were tested and β-actin was tested as control. ImageJ2x was used to conduct semi-quantitative analysis. The test was performed as mentioned above.

### Proliferation assay

The viability of the four groups of HEK293 cells were evaluated by Cell Counting Kit-8 (CCK-8) assay (keyGEN, Nanjing, China). After being treated for 36 h, the cells were seeded in a 96-well plate at a density of 1 × 10^4^ cells/well, and cultured for 24 h. CCK-8 was added to each well at a final concentration of 0.5 mg/mL for 4 h. The amount of the CCK-8 formazan dye generated by dehydrogenases in cells is directly proportional to the number of living cells. The optical density of formazan was subsequently quantified by determining the absorbance at 450 nm using a microplate reader. The experiment was performed in triplicate.

### Cell cycle analysis

We used a cell cycle assay kit from keyGEN. Briefly, 5×10^5^ cells were transfected with the plasmid in a 6 well plate. Then, after culture for 48 h in medium containing 5% FBS, the cells were harvested in PBS, washed and resuspended in PBS at 1–2×10^5^/mL, fixed with 70% ethanol to make cells permeabilised, incubated with RNase A at 37°C for 30 min, and incubated with 400 μL mixed liquid of propidium iodide and PBS at 4°C for 30 min. Cells were then analyzed by flow cytometry. At least a minimum of 1000 cells was analyzed per experiment. The experiment was performed in triplicate.

### Statistical Analysis

Statistical significance was determined using the independent sample t test. All the experiments were repeated three times.

## Results

### Clinical features of hearing loss in this pedigree

**Fig 1:**
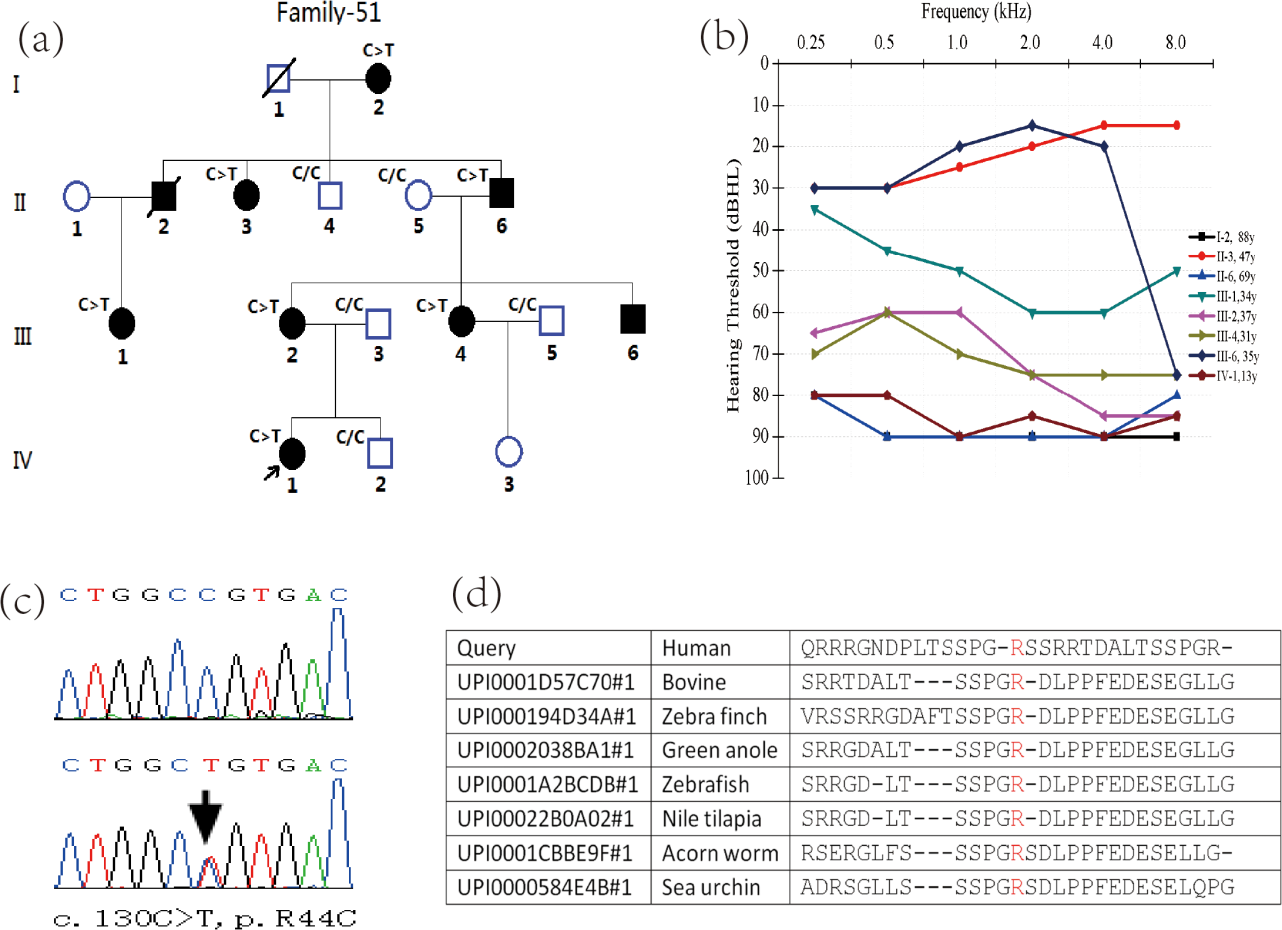
Variant analysis of MCM2. (a) Pedigree of Family #51 with hearing loss. This pedigree demonstrates nonsyndromic hearing loss of autosomal dominant inheritance. Open symbols, unaffected; solid symbols, affected. Squares, male; circles, female; slashed, deceased individual. Slanting arrow, the proband. Family members with the C>T symbol denote c.130C>T variant in *MCM2*, those with the C/C symbol had no variant in *MCM2*, and those without the symbols were not examined. Segregation of hearing loss with the c.130C>T variant in *MCM2* is remarkable in this pedigree. (b) Results of pure tone audiometry for eight members of Family #51 with hearing loss. Threshold data were obtained from the worse ears of the eight affected family members. We can clearly deduce from this graph that hearing loss is variable and mild to severe in degree in these cases. (c) A heterozygous c.130C>T variant in exon 2 of the *MCM2* gene was identified in the affected members in this pedigree. (d) Conservation analysis showed that Arg44 in human MCM2 is conserved across human, bovine, zebra finch, green anole, zebrafish, nile tilapia, acorn worm, sea urchin lineages.

Family #51 is a large Chinese family spanning four generations and characterized by hearing loss of autosomal dominant inheritance (Fig 1A). Eight living family members ranging from 13 to 88 years of age were diagnosed as having sensorineural hearing loss using pure tone audiometry tests. In most cases, the reported onset of hearing loss varied even to decades of life with subsequent gradual progression from mild to profound hearing loss involving high frequencies or all frequencies. There were no abnormalities other than hearing loss in these patients. The earliest clinical evidence of hearing loss in this family was obtained from individual IV-1 (the proband) at 13 years of age. Two members (II-3 and III-6) had no subjective hearing problems, but pure tone audiometric tests were abnormal. In all cases, hearing loss was postlingual, symmetric, and variable but mostly mild to severe in degree (Fig 1B). None of the affected individuals complained of any vestibular symptoms, and vestibular functional tests were therefore not performed.

### A missense variant in *MCM2* gene in patients in this pedigree

We then performed whole exome capture sequencing for two affected individuals (family members III-1 and III-2) to identify the disease causing gene in this family. The reads were first aligned with the human genome reference sequence (UCSC hg19) using the Burrows-Wheeler Alignment (BWA) tool. Variants (SNPs and indels) were called with vcftools of SAMTools software [25], and SNPs with a read coverage ≥4× and quality score ≥20× were considered in the initial analysis. A total of 65,271 SNPs (31,671 SNPs in family member III-1, 33,600 SNPs in family member III-2) and 4504 indels (2465 indels in family member III-1, 2039 indels in family member III-2) were identified for further analysis. Common variants were filtered out, because the disease causing variant should be rare in the population. Thus, variants found in the dbSNP database, yhSNP database (*http*:*//yh*.*genomics*.*org*.*cn/*) and the 1000 Genome SNP database were excluded. As expected, most variants identified in the two patients were indeed common. Variants located outside the coding regions were then excluded. A total of 307 candidate variants in family member III-1 and 382 candidate variants in family member III-2 were identified in the entire exome. Based on the fact that family members III-1 and III-2 should share the same causative variant, we finally found out 38 shared qualified variants. We then used the SIFT and PolyPhen-2 website to predict the functional changes in the 38 candidate variants and screened out 10 point variants.

The 10 candidate variants were examined by PCR sequencing in 13 biological family members in Family #51. Segregation of the 10 missense variants was examined within this pedigree, and only one variant in exon 2 of *MCM2* gene (c.130C>T, p.R44C, Fig 1C) segregated perfectly with hearing loss (Fig 1A). This variant was not detected in the three family members (family members II-5, III-3 and III-5) coming from outside of the family by marriage, in 145 normal controls or in 76 patients with sporadic hearing loss, suggesting that this variant is pathogenic to this pedigree. The arginine residue at the 44th position is highly conserved in most vertebrates (PolyPhen-2, *http*:*//genetics*.*bwh*.*harvard*.*edu/pph2/index*.*shtml*; Fig 1D). Therefore, we considered that this novel missense variant in *MCM2* was responsible for the autosomal dominant deafness in this pedigree.

### Presence of MCM2 in cochleae of guinea pigs

Whether *MCM2* gene is expressed in the cochlea is a fundamental issue when evaluating the role of variant MCM2 in this pedigree. The total RNA samples from guinea pig cochleae without volutes were used for RT–PCR analysis to examine MCM2 mRNA in the cochleae. The amplification of a 596 bp fragment near the 3′ end of the coding region in MCM2 mRNA was confirmed by PCR direct sequencing (data not shown), suggesting the expression of *MCM2* gene in cochleae. Transcriptome analysis of 2000 inner hair cells and outer hair cells from adult mouse cochleae also showed the expression of *MCM2* at a relatively low but stable level in these cells [24].

**Fig 2:**
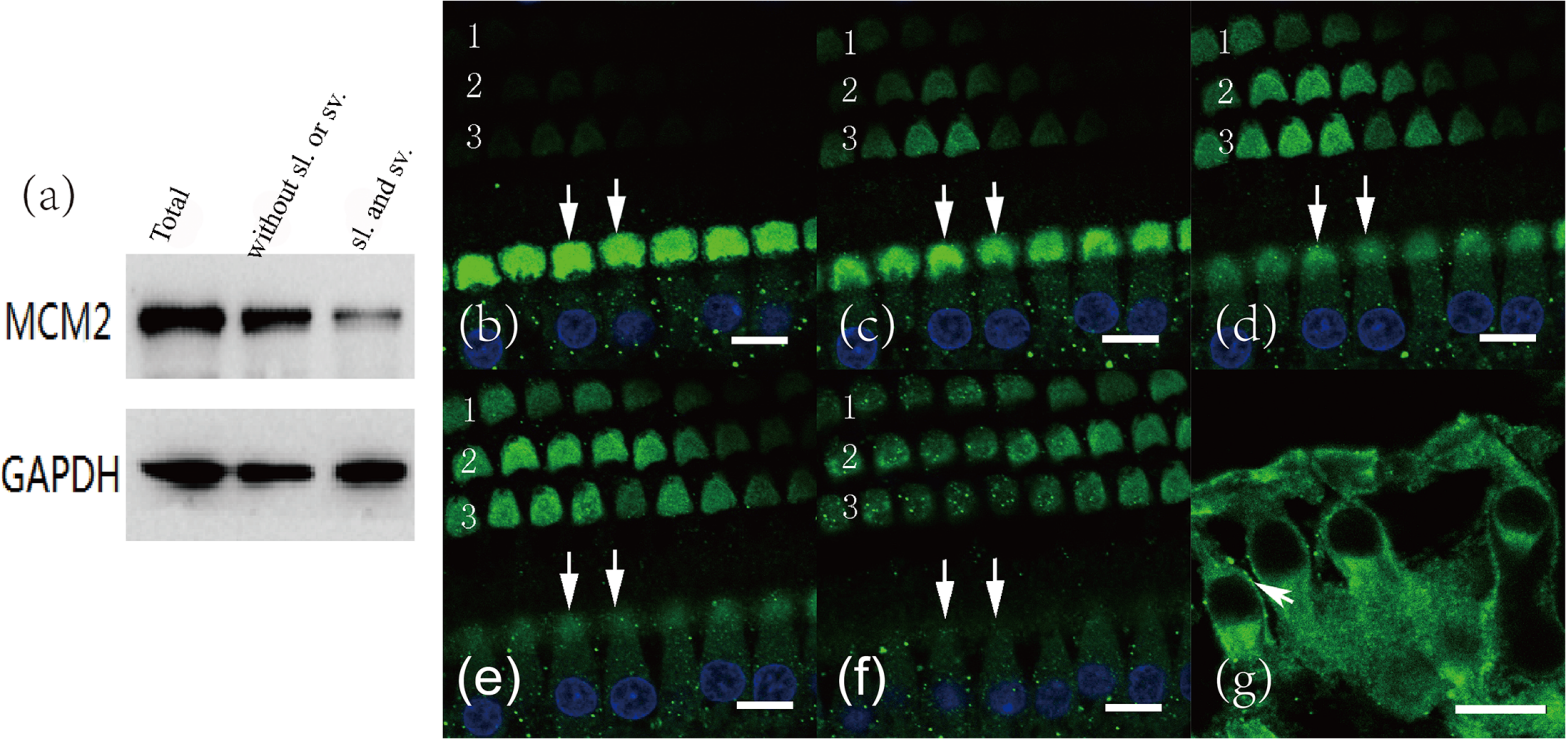
MCM2 expression in inner ears. (a) Western blot of cochlear tissues from guinea pigs. The three lanes were total cochlear protein (Total), cochlear tissues without spiral ligament or stria vasculari (without sl. or sv.), and spiral ligament and stria vascularis parts (sl. and sv.), respectively. GAPDH (glyceraldehyde-3-phosphate dehydrogenase) was used as the reference. (b-f) Whole mounts of the hearing sensory epithelial layer from mature rat were prepared for immunofluorescence using anti-MCM2 antibody. Serial sections of confocal microscopic scanning were taken from superficial to deeper layers with an interval of 1 μm/layer at the site of one line of inner hair cells (double arrows) and three lines of outer hair cells (1, 2, 3 in the panels). MCM2 was stained with Alexa Fluor 488 and nuclei with DAPI. The nuclei of the outer hair cells are not shown as they lie deeper than the level of section G. MCM2 was distributed in the cytoplasm and was highly expressed in the hair skin plate of the two kinds of hair cell. Scale = 10 μm. (g) Cochlear frozen sections from mature rat were prepared for immunofluorescence using anti-MCM2 antibody. MCM2 was stained with Alexa Fluor 488. Three outer hair cells on the left and one inner hair cell on the right were shown in the panel. Scale = 10 μm.

The protein lysates extracted from the cochleae of two guinea pigs were assayed for MCM2 protein by western blot using rabbit anti-MCM2 antibody. After removal of the cochlear modiolus and basilar membrane, which have a higher expression of MCM2, the spiral ligament and stria vascularis were also assayed for MCM2 protein. The results indicated that MCM2 was widely expressed in cochlear cells (Fig 2A). To examine the detailed distribution of MCM2 in the cochlea, whole-mount preparation of hair cells and serial section of cochlea were made for immunofluorescence using two kinds of anti-MCM2 antibody. The results showed that MCM2 is mainly expressed in the cytoplasm, especially in the hair skin plate of both inner hair cells and outer hair cells (Fig 2B–2G).

### Changes in HEK293 cells expressing p.R44C variant MCM2

**Fig 3:**
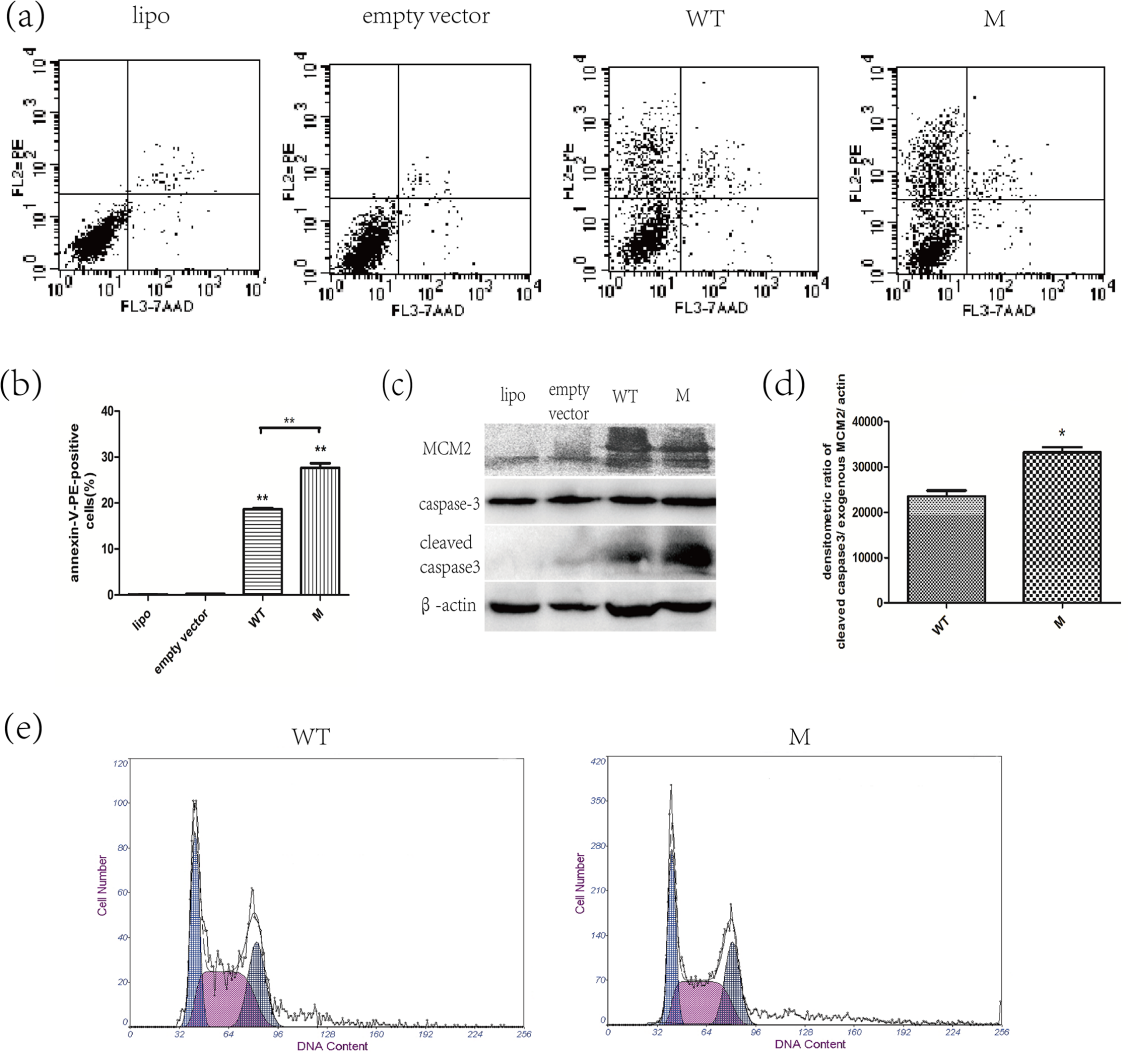
Changes in HEK293 cells expressing p.R44C variant MCM2. (a-b) HEK293 cells were treated with liposome transfection reagent only (lipo), empty vector transfection (empty vector), wild-type MCM2 plamid transfection (WT), and variant MCM2 plasmid transfection (M) respectively. After transfection for 48 h, apoptotic cell ratios were determined with annexin-V-PE-staining. Data represent the mean and SD of 3 experiments. The asterisks (**) indicate significant differences between the control and experimental groups or differences between the WT group and the M group (**p<0.001). (c-d) The western blot analysis of whole cell lysates from HEK293 cells showed a level of endogenic expression of MCM2 (as shown in lipo and empty vector groups). The upper bands in lane 3 and 4 (from the left) represent the transfected exogenous MCM2 with a GFP tag. The endogenous level of MCM2 is shown in the lower band. The ratio of cleaved caspase3 densitometry to exogenous MCM2 densitometry is compared. In the histogram, whole cell lysates from the variant MCM2 (M) transfected cells showed an upper regulated expression of cleaved caspase-3 compared with wild-type MCM2 (WT) transfected cells. Data represent the mean and SD of 3 experiments. The asterisks (*) indicate significant differences between the WT group and the M group (*p<0.01). (e) Cell cycle phases of HEK293 cells transiently transfected with wild-type or c.130C>T variant MCM2 cDNA plasmid for 48 h. Cell cycle phases were measured by flow cytometry using a cell cycle assay kit. In cells transfected with wild-type MCM2 cDNA plasmid, G1, S, and G2 phases were 27.08±4.99, 50.69±2.14, 22.21±6.04, respectively; N = 3. In cells transfected with the variant MCM2 cDNA plasmid, G1, S, and G2 phases were 28.61±1.29, 44.80±0.64, 26.57±0.80, respectively; N = 3.

To evaluate the effect of the p.R44C variant in MCM2 protein, we observed the changes in apoptosis, proliferation ability and cell cycle phases in HEK293 cells transiently expressing variant MCM2. After transfection for 48 h, HEK293 cells expressing variant MCM2 (M group) contained 27.66±1.745% apoptotic cells. In contrast, HEK293 cells expressing wild-type MCM2 (WT group) contained 18.66±0.470% apoptotic cells. The control groups that were treated with transfection reagent only (lipo group) or empty vector (empty vector group) transfection showed a low level of apoptotic cells less than 1% suggesting that the wild-type MCM2 also has a pro-apoptosis effect in HEK293 cells (p<0.001, Fig 3A and 3B). Therefore, high level of MCM2 will affect HEK293 cell apoptosis and variant MCM2 will increase the apoptosis of HEK293 cells even more significantly (p<0.001). In the western blot analysis, similar results were revealed. Since the cleaved caspase3 was not detected in the endogenous expressed wild-type MCM2 as is shown in the control groups, semi-quantitative analysis was conducted by comparing the densitometric ratio of cleaved caspase3/ exogenous MCM2/ actin in the WT group and the M group. The cleaved caspase-3 was obviously increased in the variant MCM2 expressed cells comparing with the wild-type MCM2 (P<0.01, Fig 3C and 3D). In addition, HEK293 cells also showed a level of MCM2 expression, but hardly lead to cell apoptosis.

Proliferation ability was evaluated by CCK-8 assay. The OD_450_ values were 1.41±0.25 and 1.36±0.19 (p>0.05) in HEK293 cells expressing wild-type MCM2 and p.R44C variant MCM2, respectively. Therefore, cells expressing p.R44C variant MCM2 may not significantly affect their proliferation.

Cell cycle phases were evaluated by measuring the DNA content of cells by flow cytometry. In the cells transfected with wild-type MCM2 cDNA plasmid, G1, S, and G2 phases were 27.08±4.99, 50.69±2.14, 22.21±6.04, respectively. In the cells transfected with the variant MCM2 cDNA plasmid, G1, S, and G2 phases were 28.61±1.29, 44.80±0.64, 26.57±0.80. There is no significant difference between the two groups (P>0.05) in cell cycle phases (Fig 3E).

## Discussion

In this study, a heterozygous missense c.130C>T variant in *MCM2* was identified in a Chinese family with nonsyndromic, postlingual, and progressive sensorineural hearing loss. The c.130C>T variant is uncommon and is considered to be the disease causing variant because it segregated in the eight living affected patients in this pedigree, but was not found in biologically unrelated family members, in 76 sporadic hearing loss patients, or in 145 healthy individuals. The variant arginine at the 44th residue of MCM2 is highly conserved in most vertebrates.

The phenotype in this pedigree is characterized by a great variation of hearing loss, a typical manifestation of autosomal dominant inheritance. The age of onset was quite variable. III-6 and II-3 only had mild hearing loss without subjective hearing problems, while most of the affected members suffered from moderate to severe hearing loss involving all frequencies (Fig 1B). We had found six patients with hearing loss at the beginning and considered that this variant in *MCM2* showed reduced penetrance after variant screening of the 13 family members. However, pure tone audiometric tests for all family members carrying this variant were abnormal. Therefore, this variant is characterized by complete penetrance and significantly variable expressivity.

MCM2 is widely expressed in all eukaryotes as a subunit in eukaryotic MCM2–7 (minichromosome maintenance) complex that plays a key role in regulation of the cell cycle [26–28], Furthermore, the MCM2 expressed in cytoplasm has an apoptosis enhanced effect in HL60 cells and BALB/c-derived 3T3 Cells [21–23]. MCM2 is expressed in both the nuclei and cytoplasm in various kinds of human cell lines and tissues (*http*:*//www*.*proteinatlas*.*org/ENSG00000073111-MCM2/subcellular*). But the expression pattern in human or mammalian cochlea was not detected before. Our data didn’t only show for the first time that MCM2 was expressed in various parts of the cochlea, but also revealed that MCM2 was mainly located in the cytoplasm of non-proliferative hair cells in murines from the immunofluorescence with two kinds of anti-MCM2 antibodies. This interesting result may be explained as follows: hair cells are terminally differentiated cells without cell cycle and nuclear MCM proteins are down-regulated and dissociated from chromatin when exiting cell cycle [29].

These patients carrying this variation presented a clinical feature of non-syndromic hearing loss. The explanation may be that cochlea is a very vulnerable organ and even a subtle increase in apoptotic susceptibility of inner ear hair cells could lead to progressive hearing loss in the MCM2 variation carriers. This phenomenon has also been identified in other genes causing non-syndromic hearing loss [3, 13, 30]. Sensorineural hearing loss caused by mitochondrial DNA mutations is nonsyndromic, although this mutation is widely expressed in various tissues besides cochleae [31–32].

We then tentatively assayed the effects of the R44C variant in MCM2 on apoptosis, proliferation and cell cycle phases. Since the terminally differentiated hair cells cannot survive in incubation medium, we preliminarily applied transient transfected HEK293 cells to detect the effects of variant MCM2. The cells expressing variant MCM2 displayed an increase in apoptosis without statistical significant changes in proliferation or cell cycle assay. Whether variant MCM2 will similarly affect the apoptosis process in terminally differentiated hair cells and leads to earlier impairment in cochlear function remains further detection. Although apoptosis of hair cells is a major cause of hearing loss in mammals [33–35], the exact relationship between variant MCM2 and patients’ auditory phenotype is uncertain. In-depth study of MCM2 physically interaction or co-expression with other hearing loss causing genes in cochlear cells may improve our understanding of the mechanism. And variant MCM2 knock-in animal models constructed in the future may help verify the relationship between variant MCM2 and phenotype of hearing loss.

Further investigation is required to study the function of MCM2 in inner ear cells. The results obtained in this work provide an interesting clue that may improve our understanding of the roles of MCM2, particularly in hearing loss.

## References

[1] Smith RJH, Shearer AE, Hildebrand MS, Van Camp G. Deafness and Hereditary Hearing Loss Overview In: Pagon RA, Adam MP, Ardinger HH et al, eds. GeneReviews(R). Seattle WA: University of Washington, Seattle, 1993.

[2] Bayazit YA, Yilmaz M. An overview of hereditary hearing loss. ORL; journal for oto-rhino-laryngology and its related specialties 2006: 68: 57–63. 16428895 10.1159/000091090

[3] Stelma F, Bhutta MF. Non-syndromic hereditary sensorineural hearing loss: review of the genes involved. J Laryngol Otol 2014: 128: 13–21. 10.1017/S0022215113003265 24423691

[4] Hilgert N, Smith RJ, Van Camp G. Function and expression pattern of nonsyndromic deafness genes. Current molecular medicine 2009: 9: 546–564. 19601806 10.2174/156652409788488775 PMC2840995

[5] Frolenkov GI, Belyantseva IA, Friedman TB and Griffith AJ. Genetic insights into the morphogenesis of inner ear hair cells. Nature reviews Genetics 2004: 5: 489–498. 15211351 10.1038/nrg1377

[6] Robertson NG, Hamaker SA, Patriub V, Aster JC and Morton CC. Subcellular localisation, secretion, and post-translational processing of normal cochlin, and of mutants causing the sensorineural deafness and vestibular disorder, DFNA9. Journal of medical genetics 2003: 40: 479–486. 12843317 10.1136/jmg.40.7.479 PMC1735525

[7] Hildebrand MS, Coman D, Yang T, Gardner RJ, Rose E, Smith RJ et al A novel splice site mutation in EYA4 causes DFNA10 hearing loss. American journal of medical genetics 2007: Part A 143A: 1599–1604. 17568404 10.1002/ajmg.a.31860

[8] Yan D, Zhu Y, Walsh T, Xie D, Yuan H, Sirmaci A et al Mutation of the ATP-gated P2X(2) receptor leads to progressive hearing loss and increased susceptibility to noise. Proceedings of the National Academy of Sciences of the United States of America 2013: 110: 2228–2233. 10.1073/pnas.1222285110 23345450 PMC3568371

[9] Zhao Y, Zhao F, Zong L, Zhang P, Guan L, Zhang J et al Exome sequencing and linkage analysis identified tenascin-C (TNC) as a novel causative gene in nonsyndromic hearing loss. PloS one 2013: 8: e69549 10.1371/journal.pone.0069549 23936043 PMC3728356

[10] Asan, Xu Y, Jiang H, Tyler-Smith C, Xue Y, Jiang T et al Comprehensive comparison of three commercial human whole-exome capture platforms. Genome biology 2011: 12: R95 10.1186/gb-2011-12-9-r95 21955857 PMC3308058

[11] Kuhlenbaumer G, Hullmann J, Appenzeller S. Novel genomic techniques open new avenues in the analysis of monogenic disorders. Human mutation 2011: 32: 144–151. 10.1002/humu.21400 21280146

[12] Sanders SS. Whole-exome sequencing: a powerful technique for identifying novel genes of complex disorders. Clinical genetics 2011: 79: 132–133. 10.1111/j.1399-0004.2010.01585.x 21087231

[13] Gao J, Xue J, Chen L, Ke X, Qi Y, Liu Y. Whole exome sequencing identifies a novel DFNA9 mutation, C162Y. Clinical genetics 2013: 83: 477–481. 10.1111/cge.12006 22931125

[14] Forsburg SL. Eukaryotic MCM proteins: beyond replication initiation. Microbiology and molecular biology reviews: MMBR 2004: 68: 109–131. 15007098 10.1128/MMBR.68.1.109-131.2004 PMC362110

[15] Tsuji T, Ficarro SB, Jiang W. Essential role of phosphorylation of MCM2 by Cdc7/Dbf4 in the initiation of DNA replication in mammalian cells. Molecular biology of the cell 2006: 17: 4459–4472. 16899510 10.1091/mbc.E06-03-0241 PMC1635350

[16] Bochman ML, Schwacha A. The Mcm2-7 complex has in vitro helicase activity. Molecular cell 2008: 31: 287–293. 10.1016/j.molcel.2008.05.020 18657510

[17] Bell SD, Botchan MR. The minichromosome maintenance replicative helicase. Cold Spring Harbor perspectives in biology 2013: 5: a012807 10.1101/cshperspect.a012807 23881943 PMC3809582

[18] Ishimi Y, Komamura Y, You Z, Kimura H. Biochemical function of mouse minichromosome maintenance 2 protein. The Journal of biological chemistry 1998: 273: 8369–8375. 9525946 10.1074/jbc.273.14.8369

[19] Burke TW, Cook JG, Asano M, Nevins JR. Replication factors MCM2 and ORC1 interact with the histone acetyltransferase HBO1. The Journal of biological chemistry 2001: 276: 15397–15408. 11278932 10.1074/jbc.M011556200

[20] Holland L, Gauthier L, Bell-Rogers P, Yankulov K. Distinct parts of minichromosome maintenance protein 2 associate with histone H3/H4 and RNA polymerase II holoenzyme. European journal of biochemistry / FEBS 2002: 269: 5192–5202. 12392551 10.1046/j.1432-1033.2002.03224.x

[21] Hasegawa M, Kurata M, Yamamoto K, Yoshida K, Aizawa S, Kitagawa M. A novel role for acinus and MCM2 as host-specific signaling enhancers of DNA-damage-induced apoptosis in association with viral protein gp70. Leukemia research 2009: 33: 1100–1107. 10.1016/j.leukres.2008.10.025 19058849

[22] Abe S, Kurata M, Suzuki S, Yamamoto K, Aisaki K, Kanno J et al Minichromosome maintenance 2 bound with retroviral Gp70 is localized to cytoplasm and enhances DNA-damage-induced apoptosis. PloS one 2012: 7: e40129 10.1371/journal.pone.0040129 22768239 PMC3387003

[23] Suzuki S, Kurata M, Abe S, Miyazawa R, Murayama T, Hidaka M. Overexpression of MCM2 in myelodysplastic syndromes: association with bone marrow cell apoptosis and peripheral cytopenia. Experimental and molecular pathology 2012: 92: 160–166. 10.1016/j.yexmp.2011.11.003 22115939

[24] Liu H, Pecka JL, Zhang Q, Soukup GA, Beisel KW, He DZ. Characterization of transcriptomes of cochlear inner and outer hair cells. The Journal of neuroscience: the official journal of the Society for Neuroscience 2014: 34: 11085–11095. 25122905 10.1523/JNEUROSCI.1690-14.2014 PMC4131018

[25] Li H, Handsaker B, Wysoker A, Fennell T, Ruan J, Homer N et al The Sequence Alignment/Map format and SAMtools. Bioinformatics (Oxford, England) 2009: 25: 2078–2079. 10.1093/bioinformatics/btp352 PMC2723002 19505943

[26] Tsuruga H, Yabuta N, Hashizume K, Ikeda M, Endo Y, Nojima H. Expression, nuclear localization and interactions of human MCM/P1 proteins. Biochem Biophys Res Commun 1997: 236: 118–125. 9223437 10.1006/bbrc.1997.6865

[27] Pacek M, Tutter AV, Kubota Y, Takisawa H, Walter JC. Localization of MCM2-7, Cdc45, and GINS to the site of DNA unwinding during eukaryotic DNA replication. Molecular cell 2006: 21: 581–587. 16483939 10.1016/j.molcel.2006.01.030

[28] Riera A, Fernandez-Cid A, Speck C. The ORC/Cdc6/MCM2-7 complex, a new power player for regulated helicase loading. Cell cycle (Georgetown, Tex) 2013: 12: 2155–2156. 10.4161/cc.25336 PMC3755055 23803736

[29] Ramnath N, Hernandez FJ, Tan DF, Huberman JA, Natarajan N, Beck AF et al MCM2 is an independent predictor of survival in patients with non-small-cell lung cancer. Journal of clinical oncology: official journal of the American Society of Clinical Oncology 2001: 19: 4259–4266. 11709570 10.1200/JCO.2001.19.22.4259

[30] Walsh T, Pierce SB, Lenz DR, Brownstein Z, Dagan-Rosenfeld O, Shahin H et al Genomic duplication and overexpression of TJP2/ZO-2 leads to altered expression of apoptosis genes in progressive nonsyndromic hearing loss DFNA51. American journal of human genetics 2010: 87: 101–109. 10.1016/j.ajhg.2010.05.011 20602916 PMC2896780

[31] Zhao H, Li R, Wang Q, Yan Q, Deng JH, Han D et al Maternally inherited aminoglycoside-induced and nonsyndromic deafness is associated with the novel C1494T mutation in the mitochondrial 12S rRNA gene in a large Chinese family. American journal of human genetics 2004: 74: 139–152. 14681830 10.1086/381133 PMC1181901

[32] Prezant TR, Agapian JV, Bohlman MC, Bu X, Oztas S, Qiu WQ et al Mitochondrial ribosomal RNA mutation associated with both antibiotic-induced and non-syndromic deafness. Nature genetics 1993: 4: 289–294. 7689389 10.1038/ng0793-289

[33] Lallemend F, Lefebvre PP, Hans G, Moonen G and Malgrange B. Molecular pathways involved in apoptotic cell death in the injured cochlea: cues to novel therapeutic strategies. Current pharmaceutical design 2005: 11: 2257–2275. 16026294 10.2174/1381612054367346

[34] Van De Water TR, Lallemend F, Eshraghi AA, Ahsan S, He J, Guzman J et al Caspases, the enemy within, and their role in oxidative stress-induced apoptosis of inner ear sensory cells. Otology & neurotology: official publication of the American Otological Society, American Neurotology Society [and] European Academy of Otology and Neurotology 2004: 25: 627–632. 10.1097/00129492-200407000-00035 15241246

[35] Op de Beeck K, Schacht J and Van Camp G. Apoptosis in acquired and genetic hearing impairment: the programmed death of the hair cell. Hearing research 2011: 281: 18–27. 10.1016/j.heares.2011.07.002 21782914 PMC3341727

